# Cognitive and neuropsychiatric impairment in cerebral radionecrosis patients after radiotherapy of nasopharyngeal carcinoma

**DOI:** 10.1186/1471-2377-14-10

**Published:** 2014-01-13

**Authors:** Xiaohong Wu, Mofa Gu, Guijuan Zhou, Xue Xu, Mengmeng Wu, Haiwei Huang

**Affiliations:** 1 Department of Neurology, First Affiliated Hospital of Sun Yat-Sen University, No. 58 Zhongshan Road 2, Guangzhou 510080, China; 2 Department of Radiation Oncology, State Key Laboratory of Oncology in South China, Cancer Center, Sun Yat-Sen University, Guangzhou, China

**Keywords:** Cerebral radionecrosis, Nasopharyngeal carcinoma, Cognitive impairment, Neuropsychiatric symptom

## Abstract

**Background:**

We sought to characterize the cognitive function and neuropsychiatric symptoms in cerebral radionecrosis (CRN) patients who have received conformal radiation for nasopharyngeal carcinoma.

**Methods:**

A total of 40 patients treated with radiotherapy (RT) that developed CRN (RT + CRN), 40 patients treated with radiotherapy that did not have CRN (RT-No-CRN), and 36 newly diagnosed untreated nasopharyngeal carcinoma patients (No-RT) were recruited. The cognitive function and neuropsychiatric symptoms were evaluated with Montreal cognitive assessment (MoCA), the mini-mental state examination (MMSE), activity of daily living scale (ADL), neuropsychiatric inventory (NPI), Hamilton depression scale (HAMD) and Hamilton anxiety scale (HAMA).

**Results:**

The RT + CRN group had the lowest mean MMSE, MoCA and ADL scores, while highest mean NPI, HAMD and HAMA scores among the three patient groups (*P* < 0.05). Thirty (75%) of the RT + CRN patients were deemed cognitively impaired by the MoCA compared with 9 (22.5%) by the MMSE (*χ*^*2*^ = 22.064; *P* < 0.001). Eighty-two percents of subject in RT + CRN group experienced neuropsychiatric symptoms within the past 4 weeks. Irritability, anxiety, depression and agitation in the RT + CRN group were of the most significantly frequent among the 3 groups.

**Conclusions:**

The CRN patients generally have manifestations in cognitive and psychological impairment, which have their typical characteristics, and should be considered in CRN treatment and rehabilitation. The MoCA classifies more CRN patients as cognitively impaired than the MMSE, justifying further studies of the MoCA as an appropriate screen for CRN.

## Background

Radiotherapy (RT) is the main treatment modality for nasopharyngeal carcinoma (NPC) and has produced excellent outcomes in terms of survival rate [1]. To reach satisfactory local control, the radiation field extends from the skull base to the lower neck and a total dose of about 70 Gy is generally required. In these cases, some important normal structures, including the brain, are exposed to the high dose radiation, and side effects are inevitable. One possible severe consequence is the occurrence of cerebral radionecrosis (CRN), which has an incidence rate of 3.6% ~ 8.3% in different reports [2,3]. CRN is usually irreversible and may progress over time [4]. The risk is highest in the first 6 months to 3 years after irradiation, but can persist for decades [5,6]. CRN is neuropathologically defined as necrosis with severe vascular lesions (stenosis, thrombosis, haemorrhage, fibrinoid vascular necrosis) [5]. Among patients after RT of NPC, the temporal lobes are most frequently involved [7]. The outcome varies from focal neurologic deficits to death. By using MRI, the lesion can be manifested as the early finger-like T2-hyperintensity area representative of reactive white matter edema and the late cyst-like changes corroborating with liquefactive necrosis and surrounding gliosis [8,9].

A major manifestation of CRN is the development of cognitive and neuropsychiatric impairment. Patients usually exhibit cognitive dysfunction ranges from inattention, impaired short term memory, bradyphrenia, to frank dementia, and are often accompanied with neuropsychiatric symptoms. Cognitive and neuropsychiatric impairment have been recognized as a significant problem in CRN patients. There is, however, a paucity of research in this population. The objective of this study was to characterize cognitive impairment and neuropsychiatric symptoms in CRN patients who have received conformal radiation for NPC, and also to develop a sensitive instrument for routine screening. In addition, it is known that cognitive and neuropsychiatric deficits are correlated significantly with the development of CRN. Therefore, it might be of clinical significance to determine the cognitive and neuropsychiatric function on the early diagnosis of CRN.

## Methods

### Patients

From January 2011 to October 2011, eighty patients voluntarily participated in this study. They were recruited from the First Affiliated Hospital of Sun Yat-Sen University and Sun Yat-Sen University cancer center in Guangzhou. These patients had received a radical course of conventional radiotherapy for NPC between 1998 and 2010. The irradiation dose was 68 to 74 Gy with a fraction dose of 2.0 Gy. RT was completed more than 1 year before the patients were recruited. Characteristics of the patients at baseline are listed in Table 1. A total of 36 newly diagnosed untreated NPC patients (No-RT) were included as control subjects. They matched the post-RT patients by some factors including age, gender and education years. Patients with brain metastasis, a second history of RT in head-and-neck cancer, history of head injury, history of concurrent chemoradiotherapy, with other neurological or mental diseases, and with impairments in vision, hearing or motor function that prevent them from participating in cognitive testing were excluded. The study was undertaken in accordance with the principles of the Declaration of Helsinki. The protocol was approved by the ethics committees of First Affiliated Hospital of Sun Yat-Sen University. The patients and their caregivers provided written informed consent.

**Table 1:** Patient characteristics

**Variable**	**No-RT**	**RT-No-CRN**	**RT + CRN**
	**(*n*** **= 36)**	**(*n*** **= 40)**	**(*n*** **= 40)**
Age (y)	45.5 ± 8.6	47.3 ± 9.4	48.3 ± 9.7
Gender (No. M/F)	26:10	29:11	24:16
Education (y)	9.4 ± 2.8	10.4 ± 2.9	10.3 ± 3.0
Age at the completion of RT (y)	-	43.5 ± 10.0	43.9 ± 9.2
Post-RT interval (y)	-	3.8 ± 2.6	4.3 ± 2.9
Total RT dosage (Gy)		70.5 ± 2.0	70.7 ± 1.6
CTCAE grade (1/2/3/4/5) (No.)			4/18/13/5/0

**Figure 1:**
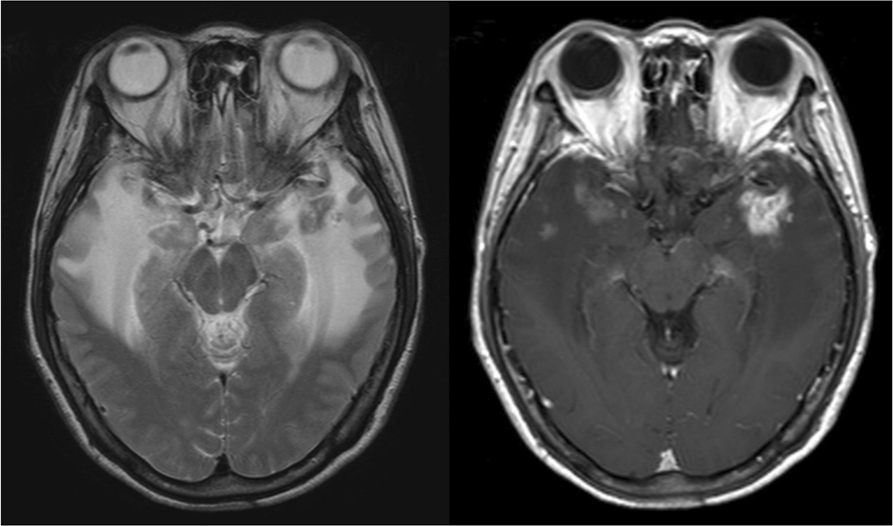
**Cerebral radionecrosis shown by magnetic resonance imaging.** T2-weighted image showing finger-like edema in both temporal lobes. Gadolinium-enhanced T1-weighted image of the same patient showing heterogeneous contrast enhancement.

Of the 80 patients, 40 showed positive CRN findings on MR imaging in the follow-up after radiotherapy (RT + CRN), with gross hypointensity in T1-weighted images, hyperintensity in T2-weighted images and heterogeneous contrast enhancement in gadolinium-enhanced T1-weighted images on either or both hemispheres, which indicated the presence of edema or cysts. Figure 1 illustrates representative images of the T2-weighted and gadolinium-enhanced T1-weighted MR images of a patient with CRN. The remaining 40 patients did not exhibit signs referable to the brain, resulting in negative findings on MR imaging (RT-No-CRN). National Cancer Institute Common Terminology Criteria for Adverse Events Version 4.0 (CTCAE v 4.0) grade was obtained from patient records [10].

### Neuropsychological testing

The Mini Mental State Examination (MMSE) [11], Montreal Cognitive Assessment (MoCA) (Beijing version) and Activity of Daily Living Scale (ADL) [12] were used to evaluate the general cognitive function. Both MMSE and MoCA were widely used cognitive screens, and MoCA has previous been documented to have superior sensitivity in brain tumor patients [13,14]. The time to administer each test was recorded. Impairment on the MMSE was defined as a score below 17 to illiterate patients, 20 to primary school culture patients, and 24 to individuals with more than 6 years of education. Cutoff scores for the MoCA was determined at 26 (scores of 25 or below will indicate impairment), as defined by the MoCA’s author [15]. A bonus point was given to individuals with <12years of education on MoCA. Neuropsychiatric inventory (NPI) [16], Hamilton depression scale (HAMD), and Hamilton anxiety scale (HAMA) were used to evaluate the neuropsychiatric symptoms. Trained research staff administered these assessment scales in counterbalanced manner.

### Statistical analysis

Descriptive statistics were calculated for all variables. For continuous variables, means and standard deviations were calculated. Descriptive statistics for categorical variables included the number and frequency in each category. Total scores for MMSE, MoCA, ADL, NPI, HAMD and HAMA were compared among the three groups using one-way analysis of variance (ANOVA) models, followed by Bonferroni test with significance level of 0.05. The chi square test was used to determine if there was a significant difference in cognitive function between the RT + CRN and RT-No-CRN group. The McNemar test was used to determine if there was a significant difference in the proportion of CRN patients deemed cognitively impaired by the MMSE and MoCA. MMSE and MoCA subdomain scores were compared between the RT + CRN and RT-No-CRN group using Wilcoxon rank sum test. Significance was defined as *P* < 0.05. The NPI frequency of symptoms was dichotomized (present or not) and compared between the three groups with Fisher’s exact test. Analyses were carried out with the SPSS, version 13.0.

## Results

### Subject characteristics

Table 1 shows the demographic and major clinical characteristics of all subjects. There was no significant difference in terms of age (F = 1.261; *P* = 0.285), gender (*χ*^*2*^ = 1.846; *P* = 0.397) and level of education (F = 1.638; *P* = 0.197) among the 3 patient groups. In addition, no significant difference was found between RT + CRN and RT-No-CRN patients in the mean total RT dosage (*t* = 0.562; *P* = 0.576), age at the completion of RT (*t* = -0.291; *P* = 0.771) and post-RT interval (*t* = -1.370; *P* = 0.172) (Table 1).

### Total scores of impairment on neuropsychological tests

The results of test are presented in Table 2. The RT + CRN group had the lowest mean MMSE and MoCA scores, while highest mean ADL, NPI, HAMD and HAMA scores among the three patient groups (*P* < 0.01). The RT + CRN and RT-No-CRN groups spent more time in completing MMSE compared with the No-RT group (*P* < 0.01), and they also spent more time in completing MoCA (*P* < 0.01). Compared to the RT-No-CRN group, the RT + CRN patients had significant high rate of cognitive impairment either deemed by the MoCA (75%, *P* = 0.001) or by MMSE (22.5%, *P* = 0.002), according to the given cutoff scores. In the RT + CRN group, thirty (75%) patients were deemed cognitively impaired by the MoCA compared with 9 (22.5%) by the MMSE (*P* < 0.001). Twenty-one (70.0%) of the RT + CRN patients with a normal MMSE were classified cognitively impaired by the MoCA. None of these patients was classified cognitively impaired by the MMSE while having a normal score on the MoCA.

**Table 2:** Total scores of impairment on neuropsychological tests

**Variable**	**No-RT**	**RT-No-CRN**	**RT + CRN**	^*^ ***p*** **Value**	^**†**^ ***p*** **Value**
	**(*n*** **= 36)**	**(*n*** **= 40)**	**(*n*** **= 40)**		
MMSE	28.9 ± 1.9	28.6 ± 1.5	25.4 ± 4.6	<0.001	<0.001
Time to complete MMSE (min)	4.0 ± 1.1	5.9 ± 1.7	5.7 ± 1.4	<0.001	0.886
MoCA	27.2 ± 2.2	27.2 ± 3.0	21.8 ± 5.3	<0.001	<0.001
Time to complete MoCA (min)	6.4 ± 1.2	8.3 ± 2.3	8.1 ± 2.0	<0.001	1.000
ADL	20.0 ± 0	20.0 ± 0	23.7 ± 9.5	0.012	0.001
NPI	3.5 ± 6.7	3.5 ± 4.5	15.6 ± 17.0	<0.001	<0.001
HAMD	4.9 ± 4.1	2.9 ± 3.0	9.7 ± 6.3	<0.001	<0.001
HAMA	5.5 ± 4.1	3.8 ± 2.8	10.7 ± 7.8	<0.001	<0.001

Data are presented as mean ± SD.

^*^Comparison between the RT + CRN group and No-RT group.

^†^Comparison between the RT + CRN group and the RT-No-CRN group.

### MMSE and MoCA subdomain scores

The MMSE and MoCA subdomain scores for the different groups were summarized in Table 3. A significant reduction in MMSE subdomain scores for registration, recall, language, attention and calculation, and orientation ability was evident in the RT + CRN group compared with the RT-No-CRN groups (P < 0.01). Consistent with the MMSE tests, the RT + CRN group had lower MoCA subdomain scores in memory and delayed recall, naming, attention, language, visuospatial/executive function, and orientation ability compared with the RT-No-CRN groups (P < 0.01).

**Table 3:** MMSE and MOCA subdomain scores in the CRN and RT-No-CRN group

**Variable**	**RT-No-CRN**	**RT + CRN**	***p*** **Value**
	**(*n*** **= 40)**	**(*n*** **= 40)**	
MMSE subdomain scores			
Orientation	10.0 ± 0	9.6 ± 1.4	0.001
Registration	3.0 ± 0	2.6 ± 0.9	<0.001
Attention and calculation	4.3 ± 1.0	3.6 ± 1.7	0.002
Recall	2.5 ± 0.8	1.4 ± 1.3	<0.001
Language	8.8 ± 0.5	8.3 ± 1.2	<0.001
MoCA subdomain scores			
Visuospatial and executive	3.8 ± 1.0	3.2 ± 1.5	0.038
Naming	2.9 ± 0.3	2.5 ± 0.8	<0.001
Attention	5.7 ± 0.5	5.0 ± 1.2	<0.001
Language	2.9 ± 0.3	2.3 ± 1.0	<0.001
Abstraction	1.6 ± 0.6	1.4 ± 0.8	0.112
Memory and delayed recall	3.5 ± 1.3	1.2 ± 1.4	<0.001
Orientation	6.0 ± 0	5.7 ± 0.8	0.001

MMSE and MOCA subdomain scores are presented as mean ± SD.

### NPI subscores

Table 4 shows the NPI frequency in three groups. Eighty-two percents of subject with RT + CRN experienced some NPI symptom within the past 4 weeks compared with 60.0% of the RT-No-CRN group (*P* < 0.01). The most frequent symptoms among the subjects with RT + CRN were irritability (87.5%), anxiety (72.5%), depression (62.5%), agitation (30.0%), apathy (30.0%) and night time behavior disturbances (15.0%). Irritability, anxiety, depression and agitation were significantly more frequent in the RT + CRN compared with the RT-No-CRN group and NPC group (*P* < 0.05). Apathy and night time behavior disturbances were significantly more frequent among the CRN compared with the RT-No-CRN group (*P* < 0.05). The RT-No-CRN group experienced more irritability than the No-RT group. Only subjects with RT + CRN had elation (8.6%), aberrant motor activity (8.6%), appetite abnormalities (8.6%), delusions (5.0%), hallucinations (5.0%) and disinhibition (5.0%).

**Table 4:** Frequency of impairment on NPI (overall and individual symptoms) in 3 groups

**Variable**	**No-RT**	**RT-No-CRN**	**RT + CRN**	^*^ ***p*** **Value**	^**†**^ ***p*** **Value**	^**‡**^ ***p*** **Value**
	**(*n*** **= 36)**	**(*n*** **= 40)**	**(*n*** **= 40)**			
Presence of symptoms in past 4 weeks, n (%)						
Any symptom	18(50.0)	24(60.0)	33(82.5)	0.354	<0.001	0.006
Delusions	0(0)	0(0)	2(5.0)	-	0.209	0.209
Hallucinations	0(0)	0(0)	2(5.0)	-	0.209	0.209
Depression/dysphoria	9(25.0)	7(17.5)	25(62.5)	0.407	0.002	<0.001
Anxiety	15(41.7)	15(37.5)	29(72.5)	0.815	0.007	0.001
Agitation/aggression	1(2.8)	3(7.5)	12(30.0)	0.620	0.003	0.009
Elation	0(0)	0(0)	3(8.6)	-	0.093	0.093
Disinhibition	0(0)	0(0)	2(5.0)	-	0.209	0.209
Irritability/liability	6(16.7)	22(55.0)	35(87.5)	0.002	<0.001	0.001
Apathy/indifference	4(11.1)	4(10.0)	12(30.0)	1.000	0.053	0.023
Aberrant motor activity	0(0)	0(0)	3(8.6)	-	0.093	0.093
Night-time behavior disturbances	2(5.6)	0(0)	6(15.0)	0.209	0.259	0.007
Appetite/eating abnormalities	0(0)	0(0)	3(8.6)	-	0.093	0.093

^*^Comparison between the No-RT group and RT-No-CRN group.

^†^Comparison between the No-RT group and RT + CRN group.

^‡^Comparison between the RT-No-CRN group and RT + CRN group.

## Discussion

In this study, we have shown that the CRN patients who have received conformal radiation for NPC generally have manifestations in cognitive impairment and neuropsychiatric symptoms, and also found some typical characteristics that have not been reported previously.

We found that the CRN patients had significant change of cognitive function, mainly reflects in short term memory, delayed recall, language, attention, orientation, visuospatial and executive function. The results of our study are similar to those of Cheung [17,18] and Hsiao [19] despite methodological differences. In these studies, RT had deleterious effects on cognitive function in NPC patients. For example, Cheung examined a group of NPC patients who had completed their radiotherapy previously, and found memory, language, motor ability, as well as executive functions were significantly impaired for those patients who developed temporal lobe necrosis after RT. Lesion volume was correlated significantly with the severity of cognitive deficits. Hsiao found that the cognitive functioning scores had significantly declined in the domains of short-term memory, language abilities, and list-generating fluency to patients at least 1year after completion of RT.

We also found a relatively high rate of general intelligence impairment among CRN patients, with 22.5% by the MMSE and 75% by the MoCA so affected. This result was inconsistent with Cheung`s study of remaining relatively intact general intelligence by the MMSE in CRN patients [18]. It is possibly related to a longer post-RT interval of mean 4.3years and bigger total RT dosage of mean 70.7 Gy in our cohort, compared with about 1 year and 58.4 Gy in the former. It has been confirmed that post-RT interval and total RT dosage are correlated with more severe CRN as well as cognitive defect respectively [20].

Compared to the MMSE, the MoCA provided more information in identification of cognitive impairment in a cohort of CRN patients, and cost only about 8 minutes to complete. It can be explained by the inclusion of subtests that measure more demanding assessment of executive function, visuospatial function, new learning, attention, and information processing speed. This is one of the few studies to assess the MoCA in NPC survivors with CRN. Because the MoCA was so well tolerated and disagreed with the MMSE in such a significant proportion of subjects, we believe that the MoCA is a more sensitive CRN-related cognitive screen suitable for clinical practice [21]. However, this study is limited by the lack of gold standard neuropsychological assessment, and a validation study further testing this preliminary hypothesis is warranted.

Another notable finding here is that CRN subjects have relatively more neuropsychiatric symptoms than those of their cerebral normal counterparts. The most frequent symptoms among subjects with CRN are irritability (87.5%), anxiety (72.5%) and depression (62.5%), with about 1 in 3 displaying agitation and apathy. Irritability, anxiety, depression, agitation, apathy and night-time behavior disturbances are significantly more frequent among subjects with CRN than those with normal MR image. Only subjects with CRN had psychotic symptoms including hallucinations and delusions.

The association between neuropsychiatric impairment and CRN has been recognized for some time, although lack of exact quantitative investigation. Nishimura reported clinical findings such as mental deterioration and motor abnormality in 12 NPC survivors with CRN [22]. Armstrong reported depression appears to increase years after RT, possibly first peaking somewhere between 4 and 6 years posttreatment [23]. Recently Tang examined a group of CRN patients with Self-Rating Anxiety Scale and Self-Rating Depression Scale, and found the patients with CRN got higher scores in both scales [24]. This result was consistent with our study of negative emotions in CRN patients.

The temporal lobes that easily involved in CRN are responsible for cognitive, emotional and psychological function. Radiation-induced damage to progenitor populations responsible for maintenance of white matter integrity and adult hippocampal neurogenesis has been suggested to play a major role in the neurocognitive impairment many cancer survivors experience [25]. The behavioral and psychological manifestations may, in part, be related to cerebral organic pathological changes (e.g., temporal lobe, limbic system, hippocampus) [26-28], and also social psychological attack (including RT adverse reaction, tumor deterioration) as well as cognitive dysfunction [29]. Post-RT patients, especially CRN victims, exhibit characteristic irritability. Though the specific cause is yet to be verified, attention should be paid to this syndrome in the long term survivors to improve their quality of life.

CRN patients who have received conformal radiation for NPC generally manifest cognitive neuropsychiatric symptoms with typical characteristics. However the study was limited by the relatively small sample size, and the lack of statistical differences found between groups (i.e. abstraction, delusion, hallucination, and aberrant motor activity) may not be a function of lack of differences, so that the results should be confirmed in some larger sample size and better match studies. Meanwhile, MoCA may be a feasible instrument for routine cognitive screening, but the sensitivity and specialty are not yet confirmed due to lack of gold standard in the study.

## Conclusions

In conclusion, we found that the CRN patients after RT for NPC have relatively high level of cognitive impairment and more frequent neuropsychiatric symptoms, which have their typical characteristics. The development of interventions to assist these persons in coping with such symptoms should also be a goal for CRN treatment and rehabilitation. The MoCA and NPI are feasible tests which are suitable for clinical practice. MoCA classifies more CRN patients as cognitively impaired than the MMSE, which led us to the hypothesis that MoCA would be a sensitive screen for CRN patients. Currently, this hypothesis still needs further study to confirm, and such study is now underway.

## Abbreviations

CRN: Cerebral radionecrosis; RT: Radiotherapy; MoCA: Montreal cognitive assessment; MMSE: Mini-mental state examination; ADL: Activity of daily living scale; NPI: Neuropsychiatric inventory; HAMD: Hamilton depression scale; HAMA: Hamilton anxiety scale.

## Competing interests

The authors declare that they have no actual or potential conflicts of interest.

## Authors’ contributions

HH made substantial contributions to the conception and design of the study, analysis and interpretation of the results, and review of the manuscript. XW substantially contributed to the conception and design of the study, data collection and analysis, interpretation of the results and drafting of the manuscript. MG performed the data analysis, participated in data collection, interpretation and preparation of the results and contributed to the drafting of the manuscript. GZ contributed to the study design, data collection and its monitoring and interpretation of the results and collaborated in the drafting of the manuscript. XX substantially contributed to the data collection and analysis, preparation and interpretation of the results. MW substantially contributed to the data collection and participated in the drafting of the manuscript. All the authors have reviewed and approved the final version of the manuscript.

## Authors’ information

We wish to highlight some relevant information about the authors: HH has a Master’s in Neurology, with vast experience in research and health service systems. XW has a Master’s in Neurology, with research interest in radiation injury. MG is an oncologist, and specializes in the treatment of nasopharyngeal carcinoma.

## Pre-publication history

The pre-publication history for this paper can be accessed here:

http://www.biomedcentral.com/1471-2377/14/10/prepub
